# Product-access challenges to menstrual health throughout the COVID-19 pandemic among a cohort of adolescent girls and young women in Nairobi, Kenya

**DOI:** 10.1016/j.eclinm.2022.101482

**Published:** 2022-06-03

**Authors:** Shannon N. Wood, Rachel Milkovich, Mary Thiongo, Meagan E. Byrne, Bianca Devoto, Grace Wamue-Ngare, Michele R. Decker, Peter Gichangi

**Affiliations:** a Department of Population, Family and Reproductive Health, Johns Hopkins Bloomberg School of Public Health, Baltimore, Maryland, USA.; b Bill & Melinda Gates Institute for Population and Reproductive Health, Department of Population, Family and Reproductive Health, Johns Hopkins Bloomberg School of Public Health, Baltimore, Maryland, USA.; c International Centre for Reproductive Health-Kenya, Nairobi, Kenya.; d Women's Economic Empowerment Hub, Kenyatta University, Nairobi, Kenya.; e Department of Sociology, Gender and Development Studies, Kenyatta University, Nairobi, Kenya.; f Technical University of Mombasa, Mombasa, Kenya.; g Department of Public Health and Primary Care, Faculty of Medicine and Health Sciences, Ghent University, Belgium

**Keywords:** Menstrual health, COVID-19, Adolescent girls and young women, Kenya

## Abstract

**Background:**

Access to menstrual hygiene products enables positive health for adolescent girls and young women (AGYW). Among AGYW in Nairobi, Kenya, this prospective mixed-methods study characterised menstrual health product-access challenges at two time points during the COVID-19 pandemic; assessed trajectories over the pandemic; and examined factors associated with product-access trajectories.

**Methods:**

Data were collected from an AGYW cohort in August–October 2020 and March–June 2021 (n=591). The prevalence of menstrual health product-access challenges was calculated per timepoint, with trajectories characterizing product-access challenges over time. Logistic regression models examined associations with any product-access challenge throughout the pandemic; multinomial and logistic regressions further assessed factors associated with trajectories. Qualitative data contextualize results.

**Findings:**

In 2020, 52·0% of AGYW experienced a menstrual health product-access challenge; approximately six months later, this proportion dropped to 30·3%. Product-access challenges during the pandemic were heightened for AGYW with secondary or lower education (aOR=2·40; p<0·001), living with parents (aOR=1·86; p=0·05), not the prime earner (aOR=2·27; p=0·05); and unable to meet their basic needs (aOR=2·25; p<0·001). Between timepoints, 38·0% experienced no product-access challenge and 31·7% resolved, however, 10·2% acquired a challenge and 20·1% experienced sustained challenges. Acquired product-access challenges, compared to no challenges, were concentrated among those living with parents (aOR=3·21; p=0·05); multinomial models further elucidated nuances. Qualitative data indicate deprioritization of menstrual health within household budgets as a contributor.

**Interpretation:**

Menstrual health product-access challenges are prevalent among AGYW during the pandemic; barriers were primarily financial. Results may reflect endemic product-access gaps amplified by COVID-specific constraints. Ensuring access to menstrual products is essential to ensure AGYW's health needs.

**Funding:**

This work was supported, in whole, by the Bill & Melinda Gates Foundation [010481].

**Research in Context**

**Evidence before the study**

Pre-pandemic evidence suggests access challenges for adolescent girls and young women (AGYW) seeking menstrual hygiene products. The COVID-19 pandemic was expected to exacerbate endemic difficulties, with experts predicting additional barriers to accessing menstrual hygiene products due to supply chain disruptions and shop closures. We searched PubMed on May 04, 2022, using search terms “(COVID-19 OR coronavirus) AND (menstrual health or menstrual hygiene or menstrual hygiene management).” This search yielded 14 articles, of which three were empirical articles focused on menstrual health among AGYW in sub-Saharan Africa. One study, from the same authorship team, presented high-level prevalence data on menstrual health product-access challenges within the context of a myriad of health and safety risks for AGYW in Nairobi, Kenya. The second study utilized content analysis to examine Kenyan primary school textbooks content on water, sanitation, and hygiene, and situated this analysis within a literature review of international menstrual hygiene management policy guidance. The third study, conducted in Zambia, reported increased uptake of free menstrual products from sexual and reproductive health hubs after they reopened from COVID-19 restrictions, with qualitative data contextualizing how COVID closures of these hubs led to ineffective menstrual product usage for AGYW. Other articles included commentaries and facility-level analyses, and studies conducted in high-income settings.

**Added value of the study**

To the best of our knowledge, this is the first study to examine menstrual health product-access challenges among AGYW in sub-Saharan Africa against the backdrop of the COVID-19 pandemic. Our mixed-methods prospective results embedded within an existing cohort of urban AGYW in Nairobi, Kenya, reveal that in 2020 over half of AGYW experienced a menstrual health product-access challenge. Six months later, in 2021, this proportion dropped to just under one in three. Both quantitative and qualitative data suggest severe financial burdens for AGYW and their families as inhibitors to purchasing and using menstrual products.

**Implications of all the available evidence**

Menstrual health product-access challenges are endemic for AGYW, however, at points of heightened COVID restrictions and economic burdens for AGYW in Nairobi, Kenya, barriers exacerbated girls’ ability to access menstrual hygiene products. These data elucidate the need to prioritize menstrual health within pandemic recovery efforts and invest in long-term economic empowerment for young women and girls.

Alt-text: Unlabelled box

## Introduction

Menstrual hygiene management is a public health issue that is gaining increasing global attention among researchers, advocates, and policymakers.^1^ While challenges to managing menstrual health occur for women over the life course, the gendered social ramifications of such challenges are profound for adolescent girls and young women (AGYW). AGYW, who are at the cusp of menarche, have less knowledge of their bodies, while concurrently navigating school, family, and dating relationships,^2^^,^^3^ with limited economic independence. Menstrual hygiene management is a multi-faceted concept that also includes issues surrounding privacy, sanitary disposal, comfort, dignity, and cleanliness, however, access to menstrual products remains a critical challenge for AGYW seeking to manage menstruation.^4^, ^5^, ^6^ Though prevalence data within low-and middle-income countries remain limited, global qualitative evidence suggests a multitude of challenges to managing menstrual health, including stigmatization of menstrual health discussions within families, schools, and social networks—without this transmission of knowledge, AGYW are left unprepared and with inadequate information to manage their menstruation.^3^^,^^7^ Economic-related challenges, including difficulty affording products and limited purchasing and negotiating power further undermine menstrual product access, including through deprioritizing young women's health and hygiene needs within families and partnerships.^7^

The menstrual health challenges AGYW face during adolescence may have a far-reaching impact on their health, and social and economic opportunities. Previous literature has explored the absence from school and/or work due to menstrual challenges.^8^^,^^9^ Specifically, Hennegan and colleagues found that among AGYW in Burkina Faso, Nigeria, and Niamey, Niger who attended school in the past year, 15-23% missed school due to menstruation over the year.^10^ Shame, taunting from peers, inadequate support from teachers and family members, and limited financial means to purchase products further exacerbate AGYW's difficulty managing menstruation.^11^ To mitigate social impact, some AGYW will find alternative products and/or rely on transactional partnerships for assistance in obtaining menstrual products.^9^^,^^12^

Prior to the onset of the COVID-19 pandemic, over half (54%) of Kenyan girls faced challenges in accessing menstrual products^13^ and an estimated 65.0% of women and girls could not afford sanitary pads.^14^ Despite efforts by the Government of Kenya to reduce prices of menstrual hygiene products, a typical pack of eight sanitary pads costs around $1.00 USD, a price unaffordable to most households.^15^ Commonly used alternatives to sanitary pads focus on natural products, including toilet paper, blankets/cloth, and other natural materials.^13^ Importantly, recent initiatives by the Kenyan Ministry of Health and Ministry of Education have sought to address barriers to menstrual hygiene management for women and girls. Specifically, in 2017, The Basic Education Act No.17 ensured provision of “free sufficient and quality sanitary towels to every girl child registered and enrolled in a public basic education institution who has reached puberty and provide a safe and environmentally sound mechanism for disposal of the sanitary towels”.^13^ Additional policies have further recognized the gender-based inequities resulting from menstrual health difficulties and sought to address knowledge- and stigma-related barriers.

In Kenya, the COVID-19 pandemic thus far has incurred profound sexual and reproductive health (SRH) consequences for AGYW^16^, ^17^, ^18^; it was expected to create additional barriers to menstrual product access, due to supply chain disruptions, restrictions on movement, and shop closures.^19^ AGYW may be particularly vulnerable to COVID-related menstrual health product-access challenges at times of heighted COVID-19 restrictions, particularly school closures, which previously served as an access point to menstrual hygiene products, given free provision of products outlined by the Basic Education Act. In March 2020, schools nation-wide closed for six months, and opened partially in October 2020 and fully in January 2021.^22^ Urban AGYW within Nairobi, Kenya are further vulnerable to these disruptions, given heightened COVID-19 cases and deaths in the capital city throughout this time period.^23^ These concerns are elevated within informal settlements in Nairobi, where AGYW are less able to practice COVID-19 preventative behaviors like social distancing and handwashing. To better understand the evolving menstrual health needs of AGYW in Nairobi, Kenya over the course of the COVID-19 pandemic, this study aimed to: 1) characterize menstrual health product-access challenges at two time points during the COVID-19 pandemic; 2) assess menstrual health product-access challenge trajectories over the pandemic; and 3) examine factors associated with menstrual health product-access challenge trajectories. We hypothesized that return to school would ease product-access challenges, however, such challenges could be sustained for AGYW with heightened economic difficulties and unable to meet their basic needs.

## Methods

This mixed-methods study draws on prospective data collected from a cohort of adolescents and young adults in Nairobi from two time points during the COVID-19 pandemic: 2020 survey wave (August–October 2020) and approximately six months later at 2021 survey wave (March–June 2021). The overarching goal of the parent study was to understand urban adolescent reproductive health behaviors; with the onset of the COVID-19 pandemic, additional objectives were added to explore the gendered impact of the pandemic on youth health and safety. Quantitative data are complemented by qualitative focus group discussions (FGDs) and in-depth interviews (IDIs) with AGYW. Baseline data are not included within the present analysis due to lack of data on menstrual hygiene. Detailed study procedures are outlined elsewhere.^16^^,^^24^

### Ethics statement

Ethical approval was obtained from the Ethics Review Committee at Kenyatta National Hospital/University of Nairobi and the Institutional Review Boards at Johns Hopkins Bloomberg School of Public Health. Informed consent was obtained from study participants prior to enrollment in the study.

### Quantitative data collection

Briefly, the youth cohort was initially recruited in June–August 2019 using respondent-driven sampling.^24^^,^^25^ At the time of recruitment, eligible youth were 15-24 years, unmarried, and residing in Nairobi County for at least one year prior to completing the baseline survey. Of those completing the baseline survey, 95% (total n=1,293; n AGYW=610) provided recontact information and consent. At the August-October 2020 wave, 94% (total n=1,217; n AGYW=610) of these participants were recontacted, consented, and completed the survey. The March-June 2021 survey wave had follow-up rate=97%; total n=1,177; n AGYW=591.

Surveys were specific to gendered impacts of COVID-19 on youth economic, health, and social experiences, including menstrual health. In adherence to COVID-19 restrictions, quantitative data were collected remotely via phone interviews using Open Data Kit (ODK) software. Surveys were conducted by trained resident interviewers in English or Swahili, based on participant preference. For the present analysis, the analytic sample was limited to AGYW who consented to be recontacted and completed surveys at both time points (n=591).

### Quantitative measures

The primary outcome of interest, menstrual health product-access challenge, was assessed via single multi-choice item at both time points^26^; this outcome is specified as a product-access challenge given it assesses only one aspect of menstrual hygiene management.^4^, ^5^, ^6^ At 2020 survey, all AGYW were asked whether they had “experienced any of the following challenges in accessing menstrual hygiene products since the start of the COVID-19 restrictions?” Responses included “could not go to the store to buy it”; “was not comfortable asking someone to go to the store”; “the products I need are not available at the store now”; “I do not have enough money to buy the products”; “other”; or “I did not experience any challenges.” At 2021 survey, young women were asked the same survey item with reference to the past six-months to capture time between survey rounds. Additionally, at 2021 survey, two further response options were included at the request of the study team and stakeholders: “I do not see the people who used to give me the products anymore” and “the organization that was supporting us with the products closed.” Per time point, a binary measure was constructed indicative of any product-access challenge vs. no product-access challenge.

A categorical measure was then created to characterize menstrual health product-access challenge trajectory, i.e., changes in product-access challenges between time points, with the following response categories: 1) no product-access challenge (did not experience challenge at either time); 2) resolved product-access challenge (challenge at 2020 but not 2021); 3) acquired product-access challenge (no challenge at 2020, but challenge at 2021); and 4) sustained product-access challenge (challenge at both 2020 and 2021).

Sociodemographic and economic factors postulated to be associated with menstrual health product-access challenge trajectories included age (16-20; 20-25 years); education (secondary or less; college/university); main activity prior to COVID-19 (student, caregiver, or other; employed in formal or informal sector); household composition (living independent of parents; living with parents); self-assessed household ladder tertile (highest; middle; lowest); prime earner in household (yes; no); and ability to meet basic needs (very able/somewhat able; not very able/not able at all). All sociodemographic and economic factors were measured at 2020 survey.

### Quantitative analysis

Descriptive analysis assessed the prevalence of menstrual health product-access challenges and specific barriers encountered, at each time point. Bivariate (not shown) and multivariable logistic regression models first examined factors associated with any menstrual health product-access challenge during the COVID-19 pandemic. Proportions of sociodemographic and economic factors by menstrual health product-access challenge trajectory were then calculated, with design-based F-statistics utilized to account for significance across trajectory category; bivariate and multinominal logistic regression examined sociodemographic and economic factors across menstrual health product-access challenge trajectories (referent no product-access challenge). Additional logistic regression models further characterized relationships between acquired vs. no product-access challenges and sustained vs. resolved product-access challenges. Covariates for the multivariable models were selected a priori based on theory, and collinearity amongst covariates assessed.

All analyses were conducted using Stata 16 (College Station, TX). Once restricted to participants who completed survey at both time points, no missing observations for the primary outcome of interest were observed; sample size floats to accommodate small amounts of missing covariate data (<1%). Sampling weights accommodate the respondent driven sampling study design using RDS-II (Volz-Heckathorn) weights, post-estimation adjustment based on 2014 Kenya Demographic and Health Survey (KDHS) population data (age, sex, education levels), and loss-to-follow-up. All presented estimates are weighted; statistical testing accounts for clustering among participants recruited by the same recruiter at baseline. Statistical significance was set a priori at p<0·05.

### Qualitative data collection and analysis

FGDs were conducted in August 2020 and IDIs at both time periods (October 2020 and April 2021). FGD participants were identified through community-partnered recruitment, with support from local youth organizations. Youth did not need to complete the quantitative surveys to be eligible for participation in FGDs. Female FGDs included approximately four participants per group (four FGDs with young women; n=16). FGDs followed a semi-structured guide, with questions focused on gender power dynamics, norms, and indirect COVID-19 impacts.

IDIs were conducted immediately following quantitative data collection at each time point with youth participants purposefully sampled from the cohort. IDI participants were selected based on age, education level, school and/or employment status, and household composition (n=10 2020; n=10 2021 IDIs with young women). Semi-structured IDIs discussed gender power dynamics and personal experiences related to COVID-19, including socioeconomic impacts and perceptions of COVID-19 restrictions.

FGDs and IDIs utilized Zoom teleconferencing. Oral consent was collected from all qualitative participants. Discussions were audio-recorded, transcribed verbatim, and translated to English language (if needed) for inductive and deductive thematic analysis. As these data focused broadly on reproductive health. Codes specific to menstrual health experiences and challenges, including COVID-related impacts, accessibility, and affordability, were extracted and organized into matrices for synthesis.

### Role of the funding source

The funding source played no role in the study design; collection, analysis, and interpretation of data; in writing the report; or in the decision to submit the paper for publication. All authors accessed the data and read and approved the final version of this manuscript and agreed to submit for publication.

## Results

### Quantitative results

At the 2020 survey, 52·0% of AGYW experienced any menstrual health product-access challenge (Table 1); six months later in 2021, this proportion dropped to 30·3%. At both time points, challenges concentrated around lack of money to buy products (45·1% 2020 versus 26·4% 2021).

**Table 1:** Menstrual health product-access challenges among young women during the COVID-19 pandemic.

	2020 Survey Wave (n= 611)	2021 Survey Wave (n= 591)
	% (n)	
**Any product-access challenge**	52·0 (318)	30·3 (179)
Specific product-access challenge*		
Lack of money to buy products	45·1 (275)	26·4 (156)
Could not go to store to buy	7·8 (48)	1·6 (10)
Was not comfortable asking someone to go to store	5·1 (31)	3·0 (18)
Products unavailable at store	3·0 (18)	0·7 (4)
Other	0·3 (2)	0·9 (5)
Organization that was supporting us closed	–	2·7 (16)
I do not see the people who used to give me products anymore	–	2·3 (14)

– not a response option during 2020 data collection.

⁎ not mutually exclusive.

Overall, 62·0% of AGYW reported a menstrual health product-access challenge at either time point (Table 2). Heightened odds of any product-access challenge throughout the pandemic were observed for AGYW with secondary or less education, compared to those in college/university (adjusted odds ratio (aOR)=2·40; 95% confidence interval (CI)=1·41-4·08; p<0·001), living with their parents, compared to those living independently (aOR=1·86; 95% CI=1·13-3·05; p=0·05), who were the prime earner in their household (aOR=2·27; 95% CI=1·17-4·38; p=0·05), and unable to meet their basic needs (aOR=2·25; 95% CI=1·41-3·59; p<0·001).

**Table 2:** Multivariable logistic regression of any menstrual health product-access challenge during the COVID-19 pandemic, compared to no challenge, by sociodemographic and economic factors at 2020 survey.

	Overall Sample Distribution	Any Access Challenge During COVID-19 Pandemic± (n=366; 62.0%)	
	Column %	Row %	aOR (95% CI)
Age			
16-20 years	29.9	65.8	ref
20-25 years	70.1	60.4	1·22 (0·61, 2·41)
Education			
Secondary or less	64.3	**69.0**	**2·40 (1**·**41, 4**·**08)**⁎⁎⁎
College/University	35.8	**49.4**⁎⁎	ref
Main activity prior to COVID-19			
Student, caregiver, and other	51.9	63.0	ref
Employed	48.1	60.9	0·81 (0·49, 1·35)
Household composition			
Living independent of parents	32.8	55.8	ref
Living with parents	67.2	65.0	**1**·**86 (1**·**13, 3**·05)*
Household SES tertile			
Highest	41.0	61.1	ref
Middle	22.8	59.2	0·78 (0·43, 1·40)
Lowest	36.3	64.8	0·83 (0·47, 1·47)
Prime earner in household			
No	85.3	60.2	ref
Yes	14.7	72.4	**2**·**27 (1**·**17, 4**·38)*
Ability to meet basic needs			
Very/somewhat able	46.6	**53.1**	ref
Not very/not at all able	53.4	**69.8**⁎⁎	**2**·**25 (1**·**41, 3**·**59)**⁎⁎⁎

Adjusted models inclusive of all variables within the table.

Bolded values indicate p<0·05.

± p-value to assess difference in any menstrual health product-access challenge across factors from design-based F statistic.

⁎ p<0.05.

⁎⁎ p<0.01.

⁎⁎⁎ p<0.001.

**Figure 1:**
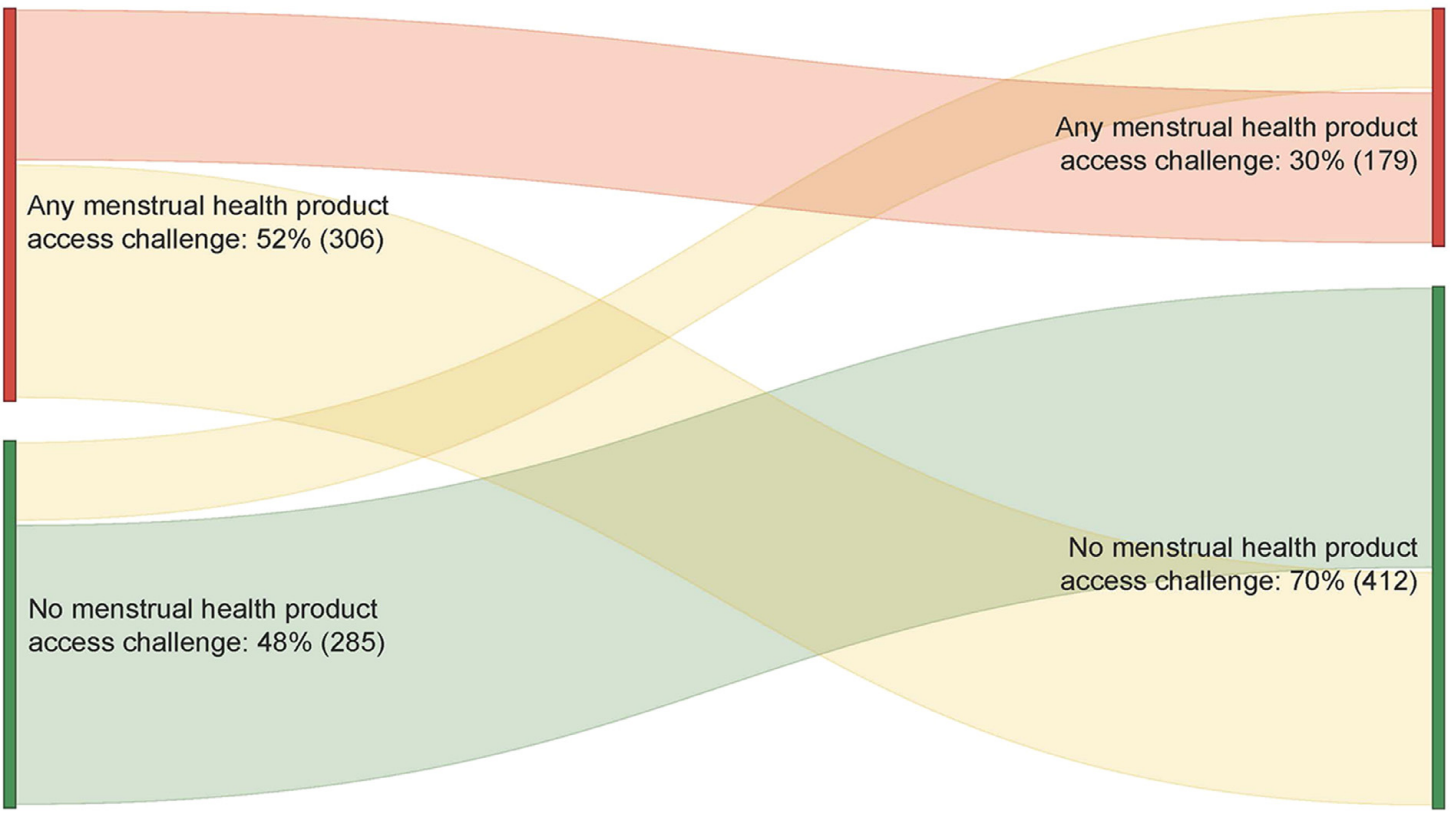
**Sankey diagram depicting changes in menstrual health product-access challenge trajectories among young women in Nairobi, Kenya from 2020 to 2021 survey waves (n=591).** Sankey diagram of transitions between any menstrual health product-access challenge and no menstrual health product-access challenge between 2020 survey and 2021 survey. Red indicates continued product-access challenges at both time points. Green indicates no product-access challenge at either time point. Yellow indicates transition from either a product-access challenge to no product-access challenge or from no product-access challenge to product-access challenge between survey waves.

When examining menstrual health product-access challenge trajectories, 38·0% of AGYW experienced no menstrual health product-access challenges and 31·7% resolved product-access challenges over the course of the pandemic, however, 10·2% of AGYW acquired menstrual health product-access challenges between 2020 and 2021 and 20·1% sustained product-access challenges across time points (Table 3; Figure 1). Bivariately, education level was associated with menstrual health product-access challenge trajectory (p<0·001), with higher proportions of more educated young women reporting no product-access challenges (50·6%) compared to lesser educated young women (31·.0%). Ability to meet basic needs was further associated with menstrual health product-access challenge trajectory (p<0·01); among young women who reported that they were very/somewhat able to meet their basic needs, higher proportions reported no product-access challenges (46·9%), compared to 30·2% of those not very/not at all able to meet their basic needs.

**Table 3:** Bivariate analysis of menstrual health product-access challenge trajectory from 2020-2021, by sociodemographic and economic factors at 2020 survey (n=591).

	No difficulty(n=225)	Resolved(n=188)	Acquired(n=60)	Sustained(n=119)	p-value±
	row %				
Total %	38·0	31·7	10·2	20·1	
Age					0.06
16-20 years	34·2	31·2	18·0	16·6	
21-25 years	39·6	31·9	6·9	21·5	
Education					**<0**·**001**
Less than secondary	31·0	35·6	11·2	22·2	
Secondary or above	50·6	24·8	8·4	16·2	
Main activity prior to COVID-19					0·09
Student, caregiver, and other	37·0	33·1	11·4	18·5	
Employed	39·1	30·3	8·9	21·7	
Household composition					0·06
Living with parents	35·0	34·1	12·3	18·7	
Living independent of parents	44·3	26·9	5·9	22·9	
Household SES tertile					0·07
Highest	39·0	32·0	14·0	15·1	
Middle	40·8	28·9	13·2	17·2	
Lowest	35·2	33·2	4·1	27·5	
Prime earner in household					0·11
No	39·8	30·9	10·8	18·5	
Yes	27·6	36·4	6·7	29·3	
Ability to meet basic needs					**<0**·**001**
Very/somewhat able	46·9	24·0	12·6	16·4	
Not very/not at all able	30·2	38·4	8·1	23·3	
Transactional relationship in past year					0·49
No transactional relationship	37·9	33·7	10·9	17·5	
Transactional relationship	38·2	28·5	9·0	24·3	

Bolded values indicate p<0·05.

± p-value to assess difference in menstrual health product-access challenge trajectory across factors from design-based F statistic.

Multivariable multinomial models are presented in Table 4, comparing resolved, acquired, and sustained menstrual health product-access challenges to no menstrual health product-access challenge (referent). Young women with secondary or lower education (adjusted relative risk ratio (aRRR)=2·73; 95% CI=1·49-5·01; p<0·001), those who were prime earner within the household (aRRR=2·36; 95% CI=1·09-5·08; p=0·02), those living with their parents (aRRR=2·12; 95% CI=1·17-3·85; p=0·05), and those who were unable to meet their basic needs (aRRR=2·82; 95% CI=1·65-4·81; p<0·001) displayed increased likelihood of resolving MHM between 2020 and 2021, compared to those with no product-access challenges. Lower education levels were similarly associated with increased risk of sustained MHM access challenges (aRRR=2·40; 95% CI=1·14-5·06; p=0·03), as was inability to meet basic needs (aRRR=2·16; 95% CI=1·21-3·85; p=0·01). Once adjusted, no sociodemographic or economic factors were significantly associated with acquiring menstrual health product-access challenges between time points.

**Table 4:** Multinomial analysis of changes in menstrual health product-access challenge trajectory, compared to those with no product-access challenges, by sociodemographic and economic factors at 2020 survey (n=591).

	MultivariableaRRR (95% CI)		
	Resolved(n=188)	Acquired(n=60)	Sustained(n=119)
Age			
16-20 years	ref	ref	ref
20-25 years	1·57 (0·74, 3·36)	0·44 (0·18, 1·11) ±	1·54 (0·59, 3·99)
Education			
Secondary or less	**2**·**73 (1**·**49, 5**·**01)**⁎⁎⁎	1·57 (0·64, 3·85)	**2**·**40 (1**·**14, 5**·06)*
College/University	ref	ref	ref
Main activity prior to COVID-19			
Student, caregiver, and other	ref	ref	ref
Employed	0·72 (0.40, 1·30)	1·27 (0·54, 2·97)	0·86 (0·42, 1·79)
Household composition			
Living independent of parents	ref	ref	ref
Living with parents	**2**·**12 (1**·**17, 3**·85)*	2·30 (0·97, 5·47) ±	1·43 (0·74, 2·77)
Household SES tertile			
Highest	ref	ref	ref
Middle	0·71 (0·36, 1·41)	0·79 (0·27, 2·31)	0·92 (0·41, 2·06)
Lowest	0·76 (0·40, 1·46)	0·30 (0·08, 1·11)	1·37 (0·67, 2·79)
Prime earner in household			
No	ref	ref	ref
Yes	**2**·**36 (1**·**09, 5**·08)*	2·11 (0·61, 7·28)	2·18 (0·93, 5·14) ±
Ability to meet basic needs			
Very/somewhat able	ref	ref	ref
Not very/not at all able	**2**·**82 (1**·**65, 4**·**81)**⁎⁎⁎	1·27 (0·49, 3·30)	**2**·**16 (1**·**21, 3**·**85)**⁎⁎

Adjusted models inclusive of all variables within the table.

Bolded values indicate p<0·05.

± p<0·10.

⁎ p<0·05.

⁎⁎ p<0·01.

⁎⁎⁎ p<0·001.

Further multivariable logistic regression models were used to examine comparisons between acquired menstrual health product-access challenge and no challenges, as well as sustained menstrual health product-access challenge and resolved product-access challenges (Table 5). Compared to those with no challenges, AGYW living with their parents reported increased odds of acquiring menstrual health product-access challenges (aOR=3·21; 95% CI=1·18-8·69; p=0·05. No further demographics were significantly associated with acquired product-access challenges nor sustained product-access challenges.

**Table 5:** Logistic regression of changes menstrual health product-access challenge trajectories (acquired vs. no challenge and sustained vs. resolved), by sociodemographic and economic factors at 2020 survey.

	MultivariableaOR (95% CI)	
	Acquired(referent= No Challenge)	Sustained(referent=Resolved)
Age		
16-20 years	ref	ref
20-25 years	0·44 (0·16, 1·16) ±	0·93 (0·38, 2·30)
Education		
Secondary or less	1·77 (0·69, 4·53)	0·91 (0·45, 1·85)
College/University	ref	ref
Main activity prior to COVID-19		
Student, caregiver, and other	ref	ref
Employed	1·09 (0·40, 3·02)	1·09 (0·52, 2·29)
Household composition		
Living independent of parents	ref	ref
Living with parents	**3**·**21 (1**·**18, 8**·69)*	0·66 (0·33, 1·34)
Household SES tertile		
Highest	ref	ref
Middle	0·89 (0·33, 2·45)	1·43 (0·61, 3·34)
Lowest	0·27 (0·07, 1·10)±	1·83 (0·89, 3·76)
Prime earner in household		
No	ref	ref
Yes	3·91 (0·84, 18·15)±	1·00 (0·43, 2·32)
Ability to meet basic needs		
Very/somewhat able	ref	ref
Not very/not at all able	1·39 (0·54, 3·59)	0·72 (0·39, 1·33)

Bolded values indicate p<0·05.

± p<0·10.

⁎ p<0·05, ^⁎⁎^p<0·01, ^⁎⁎⁎^p<0·001.

### Qualitative results

At 2020, AGYW shared that their parents typically procured sanitary pads. However, due to COVID-related loss of income, households were forced to reprioritize spending on basic needs, such as food, with menstrual hygiene products becoming a lesser priority.

Okay, right now something which the youth need are pads. Now you see, poverty is the one which parents provide sanitary pads; you see it has become hard with that story.

-Young woman, 20-24 years.

AGYW further described that loss to household finances was compounded with closures of NGOs that would generally offer free sanitary pads. These closures left young women without reliable sources and inadequate funds to obtain menstrual hygiene products.

You find jobs have been terminated and then adolescent girls have been challenged. As you can find like in slums most of them are dependent on those NGOs, so as to get pads, but you find right now they are suffering a lot because most of them have been closed.

Young woman, 15-19 years.

In cases where their parents were unable to afford sanitary pads, AGYW themselves or their peers were forced to seek out other sources.

Sometimes my mother does not get money. So, sometimes if I want to buy pads… Now, it is not there. So, you see that if you ask mum for the money, she does not have… I am made to look from outside.

Young woman, 23 years

Several AGYW participants noted that lack of money for menstrual hygiene products could lead to sexual relationships that included differential power dynamics and/or the exchange of money or goods, and accompanying sexual health concerns, particularly STI/HIV risk.

[Young women] find it is hard to get pads – then this makes them get into relationships, which they will depend on. So, when they get in [these] relationships, they start to have that early sex. You see? As like it is not anything they want. Their background pushes them so much.

-Young woman, 20-24 years

## Discussion

These mixed-methods results elucidate the product-access challenges that AGYW in urban communities of Nairobi, Kenya faced when attempting to secure menstrual products throughout the COVID-19 pandemic. In line with our initial hypotheses, difficulties in securing menstrual hygiene products dropped by 20% between survey rounds, however, approximately one in three AGYW still reported difficulties managing their menstruation at 2021 wave; sustained (20·1%) and acquired (10·2%) menstrual health product-access challenges can undermine girls’ educational and professional aspirations. At both time points, product-access challenges were primarily financial—these results challenge supply chain concerns and instead center the menstrual health product-access narrative on strained financial resources and deprioritization of menstrual health during economic and health crises. Ultimately, results corroborate endemic difficulties that women and girls face when seeking to manage their menstruation—while this analysis focused on product-access challenges at multiple points during the COVID-19 pandemic, such discussed challenges are in line with pre-pandemic difficulties^13^^,^^14^ and not unique to times of public health emergencies.

Notably, AGYW's menstrual health difficulties centered around lack of money to buy products at both time points. This was the largest single challenge indicated, with little overlap with other responses. Similarly, AGYW unable to meet their basic needs reported highest odds for any menstrual health product-access challenge during the pandemic (aOR=2.25; p<0.001) and highest risks for sustained product-access challenges across survey rounds (aRRR=2·16; p=0·02). Similar COVID-related product-access issues surrounding financial difficulties in affording products have been previously reported in Zambia and the United States.^20^^,^^21^ The greatest challenge in interpreting these results is the lack of clarity in the current study regarding the onset of menstrual hygiene product-access challenges, particularly given pre-COVID evidence indicating major difficulties in affording menstrual hygiene products.^13^^,^^14^ Government COVID-19 response policies must necessitate menstrual products as a basic need and include provision of free menstrual products within pandemic mitigation and recovery efforts. Together, these results challenge a narrative that has centered on supply chain menstrual hygiene product concerns during COVID-19 and point to economic barriers that were widely reported prior to COVID-19.

Correspondingly, reprioritization of household spending during COVID-19 directly impacted the SRH needs of AGYW, as evidenced by qualitative data describing families no longer being able to afford young women's and girls’ menstrual hygiene products. Quantitative data echoed these findings—AGYW who indicated they were the primary earner within the household had over a two-fold likelihood of resolving their menstrual hygiene product-access challenges between mid- and late-COVID periods. These results highlight the severe economic shock experienced by AGYW and their families as a result of COVID-19 and the cascade impact on AGYW's health and sanitation needs. While difficulties affording menstrual products are endemic for AGYW, these COVID-related stressors may make it more difficult for some young women to shift their recovery trajectories both immediately and longer-term. Prioritizing economic recovery, job creation, and training for women and girls, as well as addressing systemic power disparities, such as the gender wage gap, may help shift some of the economic and social power imbalances incurred throughout the pandemic.

These results further highlight that AGYW in need of money are seeking their own sources, potentially at the detriment of their health and safety. While many young women do not disclose the means that they go to seek their menstrual hygiene products, a few AGYW indicated the potential role transactional relationships played in obtaining products. Previous literature has linked money for menstrual products to transactional sex,^9^^,^^12^ and similarly elucidated sexual health and safety risks that could be incurred. Given similar sociocultural linkages between menstrual health and SRH, addressing risks for both SRH and menstrual health in a more synergistic manner could help mitigate the harmful impact of both.^27^

Qualitative data further described closure of access points as a key hindrance for AGYW attempting to access menstrual hygiene products. Counter to our initial hypotheses, AGYW did not discuss the closures of schools as an increased product-access difficulty; instead, closures of NGOs that provided free sanitary products pre-COVID were described qualitatively as a major barrier to continued, affordable access. Further research should examine convenient, community-based access points for AGYW, particularly as girls transition out of primary schools.

Results also shed light on the resilience of AGYW throughout the COVID-19 pandemic. Specifically, results highlight that nearly one in three (31·7%) AGYW resolved menstrual health product-access challenges over the course of the pandemic, including AGYW unable to meet their basic needs (aRRR=2·82; p<0·001); while these results appear counterintuitive in light of other findings on economic challenges, it is possible that ability to meet basic needs, measured at 2020 survey, concurrently resolved with menstrual hygiene difficulties. Similarly, those living with their parents (aRRR=2·12; p=0·05) displayed heighted likelihood for resolving menstrual health product-access challenges—reprioritization of household spending could have occurred after 2020 data collection. Further, some groups indicated decreased risk of sustained menstrual health product-access challenges over the course of the pandemic, including AGYW with higher education levels. Such results are promising when considering education as an access point to increased economic standing, thus addressing other potential risks, however, warrant further monitoring as the pandemic continues to unfold.

These results are not without limitations. While the pre-existing cohort of young men and young women was focused on reproductive health and family planning behaviors, baseline (pre-pandemic) data were not collected specific to menstrual health. Pre-pandemic information is necessary to fully disentangle the COVID-specific issues from endemic barriers to products in this setting. Further details surrounding recent menstruation and type of menstrual hygiene product used were not available, and two additional response options were added at 2021 survey wave. The primary outcome of interest, menstrual health product-access challenge, only captures one aspect of menstrual hygiene management and does not include other important components, such as supportive infrastructure and soap/water usage.^4^, ^5^, ^6^ Further, all independent variables were measured at 2020 survey, limiting our ability to examine sustained COVID-related risks which may impact menstrual health, specifically inability to meet basic needs. Smaller subgroups may have had limited power to detected significant changes (i.e., the acquired group). Qualitative data were not linked with quantitative data, as inclusion in the cohort was not an eligibility criterion to FGDs, and qualitative data collection covered a wide-range of adolescent SRH needs during the COVID-19 pandemic, limiting depth of analysis specific to menstrual health. Additional data types, such as those examining the supply chain, would be valuable beyond self-report and could assist in interpretation. Results may not be generalizable to Kenyan AGYW outside of Nairobi's urban communities.

The Kenyan Ministry of Health Menstrual Hygiene Management Policy is a pivotal framework for guiding AGYW's health and sanitation needs by addressing knowledge, stigma, and water sanitation and hygiene needs, with an emphasis on how to decrease these barriers for AGYW within schools.^13^ Notably, pre-pandemic product-access challenges were immense^13^^,^^14^—the COVID-19 pandemic may prove an instrumental policy window to act on endemic gender and health inequities. Additional policies can be enacted to be responsive to the current economic crisis unfolding for AGYW in Nairobi and across Kenya. Accordingly, addressing economic-related access difficulties must be a top priority to ensure girls are not further kept out of schools and the workforce as a result of managing their menstruation during the COVID-19 pandemic and moving forward. Free dignity kits, provided within communities and close to homes can ensure AGYW are able to access products in a convenient manner even during pandemic restrictions and in a way that does not incur shame or stigma. Further provision of economic incentives for menstrual health can ensure AGYW are able to choose products that best fit their lifestyles. The gendered implications of the pandemic will be far-reaching—biological vulnerability and gaps in access to essential products should not keep AGYW from reaching their full potentials.

## Contributors

Shannon N. Wood: literature search, data analysis, data interpretation, writing

Rachel Milkovich: literature search, data analysis, data interpretation, writing

Mary Thiongo: study design, data collection, data interpretation, writing

Meagan E. Byrne: study design, figures, data interpretation, writing

Bianca Devoto: study design, data interpretation, writing

Grace Wamue-Ngare: study design, data interpretation, writing

Michele R. Decker: study design, data interpretation, writing

Peter Gichangi: study design, data collection, data interpretation, writing

All authors read and approved the final version of this manuscript.

## Declaration of interests

No conflicts of interest to disclose.

## Data Sharing statement

Quantitative data are available upon request from pmadata.org. Qualitative data are not available to maximize participant confidentiality. Please, contact the corresponding author for any query.
